# Willingness to pay for one quality-adjusted life year in Iran

**DOI:** 10.1186/s12962-019-0172-9

**Published:** 2019-02-28

**Authors:** Najmeh Moradi, Arash Rashidian, Shirin Nosratnejad, Alireza Olyaeemanesh, Marzieh Zanganeh, Leila Zarei

**Affiliations:** 1 Health Management and Economics Research Center, Iran University of Medical Sciences, Tehran, Iran; 2 School of Public Health, Tehran University of Medical Sciences, Tehran, Iran; 3 Iranian Center of Excellence in Health Service Management, Tabriz University of Medical Sciences, Tabriz, Iran; 4 Health Economics Department, National Institute for Health Research, Tehran University of Medical Sciences, Tehran, Iran; 5 Health Insurance Office, Ministry of Health and Medical Education, Tehran, Iran; 6 Health Policy Research Center, Institute of Health, Shiraz University of Medical Sciences, Shiraz, Iran

**Keywords:** Willingness to pay, Quality-adjusted life year, Cost-effectiveness threshold value, WHO threshold value

## Abstract

**Background:**

Recent years have witnessed a strong tendency to apply economic evidence as a guide for making health resource allocation decisions, especially those related to reimbursement policies. One such measure is the use of the cost-effectiveness threshold as a benchmark. This study explored the threshold for use in the health system of Iran by determining society’s preferences.

**Methods:**

A cross-sectional household survey based on the contingent valuation method was administered to a representative general population of 1002 in Tehran, Iran from April to June 2015. The survey was intended to estimate the respondents’ willingness-to-pay (WTP) preferences for one quality-adjusted life year (QALY) gained. The valuation scenarios featured 12 vignettes on mild to severe diseases that can change people’s quality of life. The mean of WTP for QALY was estimated using different health instruments, and the determinants of such willingness were analyzed using the Heckman selection model.

**Results:**

WTP for QALY varied depending on the severity of a disease and the instrument used to determine health preferences. Mean low health state value were associated with high valuation. The best estimated WTP values ranged from US$1032 to US$2666 and 0.22–0.56 of Iran’s local gross domestic product (GDP) per capita in 2014. Except for educational level, significant variables differed across different disease scenarios. Generally, a high health state valuation for target diseases, high income, high educational level, and being married were associated with high WTP for QALY.

**Conclusion:**

From the general public’s perspective, the monetary value of QALY for mild to severe diseases with no risk of death was less than one GDP per capita. Therefore, the obtained valuation range is recommended as reference only for the adoption of interventions designed to improve quality of life. Future studies should estimate the threshold of interventions for life-threatening diseases or formulate transparent policies in such contexts.

**Electronic supplementary material:**

The online version of this article (10.1186/s12962-019-0172-9) contains supplementary material, which is available to authorized users.

## Background

Decision making on health care resource allocation is an overly complicated and challenging process because of the complexity of health care systems, the direct and indirect effects of a policy decision on other divisions of such systems, and the scarcity of health care resources, particularly financial resources. In view of resource constraints, health care systems often tend to make decisions that maximize people’s health at the highest efficiency. To achieve these goals, decision makers have employed different criteria and decision analytical tools, such as health economic evaluation [1–3]. Health economic evaluation plays a valuable role in the establishment of health care priorities by assisting decision makers in allocating limited health care resources to interventions that present the greatest health gain for an entire society [4]. The most common type of health economic evaluation is cost-effectiveness analysis (CEA), which evaluates alternative interventions in terms of their differences in cost and quality-adjusted life year (QALY) gained [5]. A major shortcoming of CEA, however, is that the result of analysis is only a numerical value (i.e., incremental cost per QALY) that indicates, in comparison with alternatives, which QALYs are cheaper. This is minimally comprehensible to policy makers and fails to offer an acceptable solution to the issue of which intervention offers the best value for money or how much cost per QALY should be acceptable and worth investing in given the resources available to a national health care system [6]. Addressing this issue necessitates establishing a cost-effectiveness (CE) threshold as a benchmark at the national level. As expected by Eichler [7], the CE threshold has increasingly emerged as a requirement in countries that have introduced economic evaluation guidelines into their health care systems and have introduced CE results as a fourth hurdle alongside efficacy, quality, and safety, in decision making regarding pharmaceutical reimbursement [8, 9]. Iran likewise exhibits a strong inclination to apply economic evidence as basis for making resource allocation decisions, especially those related to reimbursement policies [10–12]. This tendency is demonstrated by the establishment of a health technology assessment office in Iran’s Health Ministry in 2007, the formulation of pharmacoeconomic evaluation guidelines by Iran’s Food and Drug Administration for the inclusion of new drugs in the national drug list, the growing number of CEA studies on medical equipment and treatments, and student admissions in the fields of health economics, pharmacoeconomics, and health technology assessment [10–12]. In practice, however, Iran applies neither a transparent decision criterion nor a CE threshold or any evidence-based approach to reimbursement decisions [13]. The only measure used in the country is the World Health Organization’s (WHO) recommendation for choosing cost-effective interventions based on a country gross domestic product (GDP) per capita. So an intervention is very cost-effective/cost-effective if the incremental cost effectiveness ratio fell below one/between 1 to 3 times a country’s GDP per capita. This threshold taken from 2001 WHO Commission on Macroeconomics and Health (CMH) report which stated “according to some estimation, each life year is valued at around three times the annual earnings”. So, based on this statement, spending per capita estimated value to achieve one extra healthy life year was reasonable [14, 15].

The simplicity and easy use of WHO criterion increase the employing it worldwide particularly by low/middle income countries. A review by leech on studies which cited the WHO EC threshold during 2000–2015 showed that 66% of studies in low-/middle income countries used it [16]. In spite of, growing use of WHO CE threshold value, due to lacks a clarification on the value derivation, the concerns around applying it in policy making context are raising [16–18]. Similarly, in Iran health system context, there is a general consensus to establish an evidence-based local CE threshold value with the aim of applying CEA results in practice to maximize health and efficiency. Given the limitations of the WHO’s recommendation and the drawbacks of other approaches to obtaining the CE threshold including the weaknesses of the league table to apply in practice and lack of explicit supported evidences in revealed past health resource allocation decisions, the stated-preference approach was chosen as the best method to explore an evidence-based CE threshold for use in Iran health care system [19]. Therefore, this study aimed to investigate Iranian preferences for monetary value for QALY through eliciting individuals’ maximum willingness to pay (WTP) for a given health utility gain and also to assess its consistency WHO CE threshold value.

## Methods

### Study design and sampling

To determine WTP for the QALY gained from interventions intended to improve quality of life, a cross-sectional household survey and face-to-face interviews were conducted with a representative general population of 1002 in Tehran, Iran from April to June 2015. The sample was selected from 22 municipal regions of Tehran via two-stage cluster sampling, with consideration for probability proportional to size sampling [20]. The inclusion criteria were being a household member aged 18–79 years old, having Iranian nationality, being able to understand and speak Farsi, and having no mental disability. For the interviews, eligible respondents were chosen randomly on the basis of the detailed protocol. The protocol of study was approved by the Ethics Committee of Shahid Beheshti University of Medical Sciences. The Verbal consent was approved by the Committee and it was obtained from participants according to a step-by step guide which was developed for that. Respondents who were reluctant to be interviewed were substituted with the respondents most similar to the hesitant individuals in terms of age and gender.

### Preference elicitation scenarios

To elicit preferences, 14 hypothetical disease scenarios were presented to 29 individuals during a pilot study, and each of the respondents was asked to value at least two scenarios. These hypothetical scenarios were disease vignettes that vary in intensity (mild, moderate, and severe) and course of a given disease over time (Table 1). They were based on empirical evidence, in which records (cards) indicate two types of information: disease labels with specific descriptions of diseases and generic descriptions of health states targeted within the framework of the five-level EuroQol five-dimension questionnaire (EQ-5D-5L) plus one extra dimension on cognitive functioning (i.e., the five-level six-dimension questionnaire or EQ-6D-5L) [21].

**Table 1:** Descriptions of disease vignettes. Source: Ref. [21]

No.	Disease	EQ-6D-5L	Course of disease over time
1	Otitis media, yearly recurrent (chronic otitis media)	112,412	Recurrent
2	Migraine, monthly recurrent	124,413	Recurrent
3	Allergic rhinitis, seasonal (hay fever), yearly recurrent	111,211	Recurrent
4	Gastritis, 6 months, untreated (inflammation of the stomach lining)	112,311	Recurrent
5	Cystitis, chronic recurrent (recurrent cystitis)	112,311	Recurrent
6	Athlete’s foot, untreated (tinea pedis)	111,211	Chronic
7	Infection of the nails, chronic, untreated, onychomycosis	111,211	Chronic
8	Eczema, chronic permanent (dermatitis atopic)	112,211	Chronic
9	Back and neck pain	322,311	Chronic
10	Depression, mild	112,131	Chronic
11	CVA/stroke, moderate impairments	333,323	Chronic
12	Dementia, severe	345,235	Chronic
13	Hip fracture	444,311	Chronic
14	Spinal cord lesion, low (stable phase)	544,321	Chronic

**Fig. 1:**
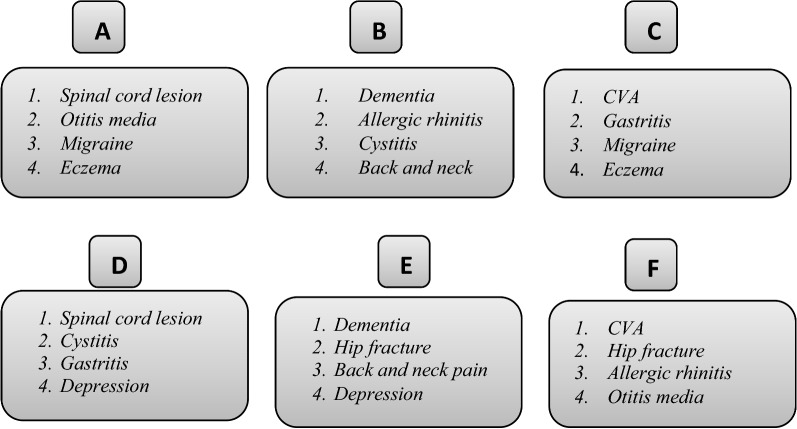
Grouping of disease scenarios

On the basis of the pilot study’s results, corrections were made to the questionnaire. For example, two out of the 14 scenarios (i.e., nail infection and athlete’s foot) were excluded because they were deemed insufficiently important, and payment questions were changed from open-ended payment questions to closed-ended ones. To increase the validity of the responses, we classified the remaining 12 disease vignettes into six groups (A–F). Each group consisted of four scenarios: one featuring permanent severe chronic disease (CVA), spinal impairment, or dementia; another featuring permanent mild chronic disease; and two others featuring recurrent mild diseases. Each scenario was indicated in two of the classification groups, as shown in (Fig. 1). Then, each classification group was attached to the questionnaire (Additional file 1), which was randomly administered to the respondents, according to the detailed protocol described in the study design and sampling section.

### Preference elicitation techniques

We used the visual analogue scale (VAS), the time trade-off (TTO) technique, and the contingent valuation method (CVM) to determine the health and WTP preferences of the respondents [22–24]. To identify health preferences, the respondents were asked to indicate their current health states by using the VAS, after which an assigned vignette was shown to the respondents, who were then asked to imagine themselves in such a situation. After this visualization task, they were asked to re-rate their health states by using the VAS. For the TTO measurement, we asked the respondents to imagine themselves having a target disease and state the maximum time that they are willing to sacrifice to avoid being in such a situation for the rest of their life. Two durations were used in this “trading” exercise: one adjusted with age-specific life expectancy and the other a fixed 10-year period [25]. Finally, the respondents were asked to indicate their maximum WTP for a hypothetical treatment that would cure them of their hypothetical disease. To reduce any biases associated with the hypothetical nature of stated WTP studies, this section was design according to key aspects of CV construction suggested by Smith -from a scenario construction to mode of administration [26]. For example, the disease scenarios were based on the patients’ experiences and among different payment vehicles, the out of pocket payment chosen as an appropriate one because it is a common form of payment in Iran health system and OOP constitute a high share of total health expenditure, insofar as, reducing it was one of the main objective of Iran health transformation plan [27] Also, the payment card (PC) method, accompanied with a follow-up open-ended question, was chosen to identify WTP [28]. Among the commonly used closed-ended questions of CVM, PC questions were chosen for this work primarily because such questions cover a diversity of diseases in each disease group, from mild to severe, which in turn, correspond to a wide range of money amounts that respondents would be willing to pay. Another reason for the choice was to avoid respondent fatigue and confusion in the valuation of the four disease scenarios; such conditions give rise to the bargaining tendency observed in the two other CVM methods (i.e., dichotomous choice and bidding game formats). To minimize biases arising from ranges, based on pilot test, we designed a range of 18 bid amounts on the basis of their appropriateness for all scenarios (The bid amounts are available in Additional file 1). The range put forward in this research was US$333 to US$83,333, which is equal to 0.07–17.5 of Iran’s local GDP per capita in 2014. So as not to constrain the respondents with our range, the “less than” and “more than” value options were incorporated into the lower and upper limits of the range. Then the follow up question was asked to determine the exact amount. The bid amounts were presented only to the respondents who were willing to pay even meager amounts to avoid health utility losses. Moreover, to minimize hypothetical bias, reminding Budget constraint and asking follow up question to identify source payment used as ex-ante and ex-post approaches, respectively. As for the individuals with zero responses, their reasons for their unwillingness to pay were ascertained.

### Data analysis

We used the chained approach in estimating the mean of WTP for one QALY in different scenarios. We then investigated the factors associated with such willingness as follows. We identified independent factors, including the socioeconomic and demographic characteristics of the respondents, the disease scenario familiarity/experience, hospitalization experience within the past year, near-death experience from a year before, and existence of a chronic disease or disability (physical or mental). The Heckman selection model was then used to probe into the factors associated with WTP for QALY [29, 30].

Data management and analysis were conducted using MS Excel 2010 and Stata/MP.

## Results

### Descriptive statistics

As previously stated, 1002 household members were interviewed using the designed questionnaire. The sample replacement was 8.2%. The mean age of the respondents was 43 years old, and 41% of them were male. The general characteristics of the sample are presented in Table 2.

**Table 2:** Sample characteristics

No. of respondents	1002
Mean age (year) (std. dev.)	43.36 (15.6)
Range (year)	18–79
Male gender (%)	408 (40.7)
Married (%)	731 (71.2)
Head of household (%)	372 (37.1)
Household size (std. dev.)	3.3 (1.2)
Education	
Elementary education ≤ 5 years	218 (21.8)
Secondary education ≤ 8 years	107 (10.7)
High school diploma ≤ 12 years	348 (34.8)
University education	328 (32.7)
Monthly household cost group (US$)	
Less than 167	248 (24.7)
167–333	473 (47.2)
333–667	232 (23.1)
667–1000	29 (2.9)
More than 1000	20 (2.0)
Hospitalization and near-death experience from a year before	
Individual experience (%)	109 (10.9)
Family member experience (%)	131 (13.0)
Near-death experience (%)	50 (5)
Having a chronic disease	
Individual experience (%)	61 (6.1)
Family member experience (%)	102 (10.2)
Concurrent	41 (4.1)

### Health and WTP preferences of respondents

The respondents’ preferences, including those for health and WTP, are shown in Table 3. The table includes the mean health state valuation derived through the VAS and TTO measurements for each disease scenario before and after the presentation of the scenarios. The respondents assigned the lowest health state value to the spinal cord lesion scenario. The scenario with the highest mean value was allergic rhinitis, followed by otitis media. The mean WTP values ranged from US$560 for otitis media to US$11,944 for CVA. Out of the 12 disease scenarios, more severe diseases (i.e., spinal cord lesion, dementia, and CVA) were assigned the lowest health and highest WTP mean values. By contrast, less severe diseases were assigned the highest health and lowest WTP mean values. Although the respondents exhibited a high positive WTP for all the disease scenarios, a zero WTP indicated that less severe diseases (e.g., allergic rhinitis, otitis media, and migraine) acquired less WTP preference. The rate of non-traders and respondents with zero WTP somewhat confirm the effect of disease severity. For example, in mild diseases such as allergic rhinitis, 72% of respondents were non-traders while in the spinal cord scenario; the number is only 3%. On the other hand, although the majority of individuals had positive WTP in diseases (82% and 93%), the mean WTP value for spinal cord is 19 times of allergic rhinitis’ value.

**Table 3:** Health and WTP preferences of respondents across different disease scenarios

Scenario	Spinal cord lesion	Depression	Eczema	Otitis media	Cystitis	Gastritis	Back and neck pain	Allergic rhinitis	Dementia	CVA	Hip fracture	Migraine
No. of respondents	334	334	334	334	323	334	334	334	334	334	334	334
VAS (current health state)	0.76	0.76	0.76	0.76	0.77	0.78	0.75	0.77	0.75	0.78	0.76	0.76
VAS (disease)	0.2	0.48	0.6	0.6	0.52	0.58	0.43	0.63	0.3	0.22	0.51	0.57
TTO-adjusted (disease)	0.49	0.86	0.95	0.96	0.9	0.92	0.89	0.97	0.51	0.52	0.87	0.93
Rate of traders in TTO-adjusted	97%	75%	37%	31%	63%	52%	59%	28%	96%	97%	63%	46%
TTO-10 year (disease)	0.47	0.89	0.96	0.97	0.91	0.94	0.89	0.97	0.51	0.52	0.88	0.94
Rate of traders in 10 year-TTO	97%	46%	20%	18%	38%	29%	43%	16%	94%	98%	43%	24%
Positive WTP	93%	90%	84%	83%	90%	87%	87%	82%	88%	92%	89%	85%
Zero WTP	7%	10%	16%	17%	10%	13%	13%	18%	12%	8%	11%	15%
Min WTP-non-zero value (U$)	15	10	10	10	15	10	13	10	10	33	17	300,000
Max WTP (US$)	166,667	43,333	10,000	16,667	25,000	16,667	16,667	16,667	233,333	116,667	25,000	83,333
Mean WTP (US$)	11,215	2013	675	560	2096	1243	977	579	7357	11,944	2178	1471

Another interesting point associated with the trading behavior of respondents to scarify time or money is people are unwilling to scarify life time particularly in mild disease states, while the money is acceptable even small amounts. An example scenario is eczema, 63% of responders were non-traders while only 16% of them have zero WTP.

### Mean WTP for QALY in 12 disease scenarios

Table 4 shows the mean values of WTP for QALY in the 12 disease scenarios. The values varied depending on the severity of a disease and the instruments used to identify health preferences. In all the health preference measures, the respondents unanimously assigned the highest WTP value for a QALY gained to the CVA, spinal cord lesion, and dementia scenarios. The lowest was accorded to the back and neck pain and allergic rhinitis scenarios. Insofar as it is necessary to select one value to apply in the health policy context, the QALY value derived with TTO is more preferable to that obtained using VAS [31]. Moreover, we believe the TTO-adjusted technique is more desirable than the 10-year TTO technique because of its conformity with individual remaining expected life time. On these bases, a range of US$1032 to US$2666 can be selected as the most suitable mean WTP for QALY—from the perspective of Tehran’s citizens. This range would reflect monetary value of one QALY gained from improvement in the quality of life among individuals suffering from chronic mild to severe diseases. The ratios of WTP for QALY in relation to GDP per capita across the different scenarios are also listed in Table 4. These values were less than the one local GDP per capita of Iran in 2014.

**Table 4:** WTP for QALY across different disease scenarios

Health instrument	Scenario											
	Spinal cord lesion	Depression	Eczema	Otitis media	Cystitis	Gastritis	Back and neck pain	Allergic rhinitis	Dementia	CVA	Hip fracture	Migraine
VAS												
WTP/QALY	1450	539	373	329	802	541	305	367	1672	1688	753	530
% GDP**	0.30	0.11	0.08	0.07	0.17	0.11	0.06	0.08	0.35	0.35	0.16	0.11
TTO-adjusted												
WTP/QALY	2102	1273	1841	1195	1921	1659	1032	1152	1667	2666	2067	1599
% GDP**	0.44	0.27	0.39	0.25	0.40	0.35	0.22	0.24	0.35	0.56	0.43	0.34
TTO 10-year												
WTP/QALY	1827	1220	634	1301	1327	838	742	606	1855	2402	1912	1306
% GDP**	0.38	0.26	0.13	0.27	0.28	0.18	0.16	0.13	0.39	0.50	0.40	0.27

* All monetary values are in US dollar in 2014

** WTP for QALY as a % of GDP per capita

For each disease scenario, regression analysis using the Heckman selection model was performed to examine WTP for QALY-related factors, including individual characteristics and health histories [29, 30]. The significant variables are reported in Table 5. The results indicated that although the variables (except educational level) varied across different disease scenarios, a high health state valuation for target diseases, high monthly household cost group (as a proxy for income), high educational level, and being married were generally associated with high WTP for QALY.

**Table 5:** Factors associated with WTP for QALY in each disease scenario

Variable	Disease											
	Spinal cord lesion	Depression	Dementia	Eczema	Gastritis	Migraine	Hip fracture	CVA	Allergic rhinitis	Back and neck pain	Cystitis	Otitis media
Income	1.04*	0.89*	–***	1.17**	–	–	0.70*	1.08*	–	–	0.52*	1.55**
Education	0.68*	0.69*	0.70*	0.87**	0.65*	0.37**	0.76*	0.90*	0.94**	0.83*	0.42**	1.14**
Marriage	0.74**	1.18*	–	–	1.29*	–	–	–	–	–	1.60*	–
Disease/health state	–	–	1.62**	4.85**	4.42*	7.29*	–	2.35**	− 9.5*	–	3.32*	–
Head of household	–	− 1.39**	–	–	–	–	–	–	–	–	–	–
Familiarity with disease	–	–	− 1.76*	-	–	–	–	–	–	–	–	–
Gender	–	–	–	–	–	–	–	–	–	–	1.17*	–
Household size	–	–	–	–	–	–	–	–	–	–	–	0.73**

* *p* value < 0.05, **p-value < 0.1, ***non significant

## Discussion

Our results showed that Iran’s general public was willing to pay for one QALY but that such willingness varied depending on disease severity and health preference instrument. Thus, more severe health states were associated with high WTP for QALY. The best estimated values ranged from US$1032 to US$2666. The lower limits of the range was obtained for the back and neck pain scenarios, followed by the allergic rhinitis scenario, and the upper limits were derived for the CVA scenario, followed by the spinal cord lesion scenario. Although the values differed depending on disease scenario, all of them were equal 0.22–0.56 of Iran’s local GDP per capita in 2014, i.e. less than the one. This is while, in the PC method, a bid range is designed to be wide enough to cover a GDP per capita of 0.07–17.5. Therefore, the method cannot drive individuals to state values lower than the aforementioned bid range.

In parallel with the present research, two studies on WTP for QALY were performed from the perspective of heart disease and diabetic patients [32, 33]. These groups were chosen because of the fact that cardiovascular diseases and diabetes are often stated as the two increasingly costly health problems in Iran, with the former being a serious disease with fatal consequences and the latter being a chronic disease with disabling complications [34]. The highest monetary values of QALY were US$3599 and US$4453 for cardiovascular diseases and diabetes, respectively. Although the mean values derived from the patients were greater than the upper value obtained from the general public [35], the patients’ preferences align with society’s preference, which is less than one GDP per capita. The elicited values raise a major issue in employing an appropriate CE threshold, that is, a policy issue related to the WHO’s recommendation on using less than three GDP per capita as a CE threshold [13]. This is the most important policy challenge in the context of Iran health system because no evidence has been provided on the link or comparison between WTP per QALY and the WHO CE threshold in the country. This points to a policy challenge that concerns the appropriateness of the WHO’s recommendation on choosing cost-effective interventions, particularly for any countries with limited resources or unsustainable such as oil-dependent economies.

More interesting, our empirical results on inappropriateness of WHO CE threshold is in the same line with a study of woods et al. on estimating relationship of countries GDP per capita and CE threshold value based on their income level. It revealed the appropriate range for low,- middle and middle,-high income countries as 0.1–0.51 and 0.18–0.71 GDP per capita, accordingly [18]. So, regarding the concerns around employing WHO recommendation in policy making context including debates on its value derivation and relationship with country GDP per capita-, developing context-specific thresholds value by national health care system through considering society priorities preferences and also examining it compared with WHO CE threshold value to establish a reasonable local CE value are recommended [16–18]. And use GDP per capita only as a criterion for updating the elicited CE threshold value.

Finally, it is essential to remind that this study investigate society preferences for interventions which improving quality of life, so the recommended range for life-saving interventions may be differ and higher. As a systematic review by Nimdet showed that the average ratio of WTP per QALY and GDP per capita for improving quality of life was 0.59, which is significantly lower than the ratio of life extension or life-saving treatments (2.03) [36].

## Conclusion

In view of the policy argument discussed above, the general public’s perspective was that the monetary value of QALY for treatments meant to improve quality of life was less than one GDP per capita. This value may differ from the value of QALY for life-prolonging or life-saving interventions because of the exclusion of the risk of death in our valuation [35]. A recent study by Viyanchi on selecting reimbursement criteria from the viewpoint of Iranian health decision makers illustrated that life-threatening conditions are the most important factors that should be considered when deciding on health insurance reimbursements before pharmacoeconomic evaluations [37]. Therefore, the range determined in the current work is recommend as a guide only for the adoption of interventions intended to improve quality of life. We suggest that other researchers carry out studies that estimate the CE threshold for interventions in life-threatening diseases or formulate transparent policies, such as the National Institute for Health and Care Excellence’s end of life policy, in such contexts [38].

## Additional files

**Additional file 1.** The English questionnaire which including disease group A.

## Abbreviations

WTP: willingness to pay
QALY: quality-adjusted life year
GDP: gross domestic product
CEA: cost-effectiveness analysis
CE: cost-effectiveness
EQ-5D: EuroQol five-dimension
VAS: visual analogue scale
TTO: time trade-off
CVM: contingent valuation method
