# Supplementary material for: Willingness to pay for one quality-adjusted life year in Iran
Source: Cost Eff Resour Alloc. 2019 Feb 28;17:4. doi: 10.1186/s12962-019-0172-9 (PMC6396529; doi:10.1186/s12962-019-0172-9)
Supplement: Supplementary file 1 — Additional file 1. The English questionnaire which including disease group A. [file 12962_2019_172_MOESM1_ESM.doc]

**Shahid Beheshti University of Medical Sciences**

**Willingness to pay for one quality-adjusted life year**

1. Municipal region No: ….. 2. Cluster No:….. 3. Respondent's No: 5

**Dear respondents,**

Hello,

The purpose of this study is “estimating the value of one year living in full health state from your perspective”. This study was undertaken as a PhD thesis at Shahid Beheshti University of Medical Sciences. The results of this study will be used in policy making for improving and increasing of society’s health, as well as distribution of scare health system resources in equity manner. Therefore, your participation will be appreciated.

We would like to ask you a series of questions about your current health state, your willingness to pay to get health benefit and also some questions about your socio-economic status and your health history.

There is no right or wrong answer to the questions, and only your personal perspective is important to us. We also assure you that all information we receive will remain completely confidential and your answers will not be shared with anyone other than the team members. Your participation in this research is completely voluntary and you can, of course, decline to, answer any question, as well as to stop participating at any time.

This interview will take about 20 minutes of your time, if you are agree, let’s begin.

**Thanks for your cooperation**

**Diseases group: A**

1) Spinal Cord Lession 2) Otitis Media 3)Migraine 4) Eczema

1. **Health utility measurement**

**A) EQ_5D**


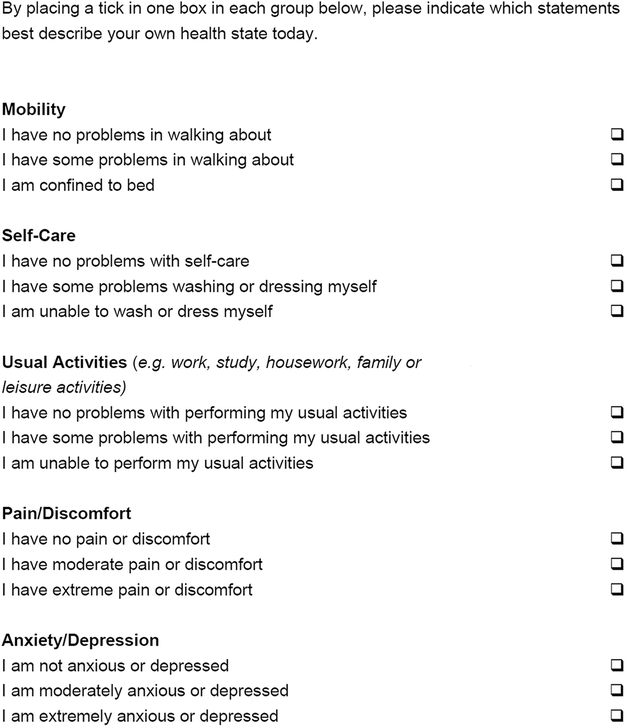


**B) VAS**

In this section, we asked respondents to indicate their current health states by using the VAS.


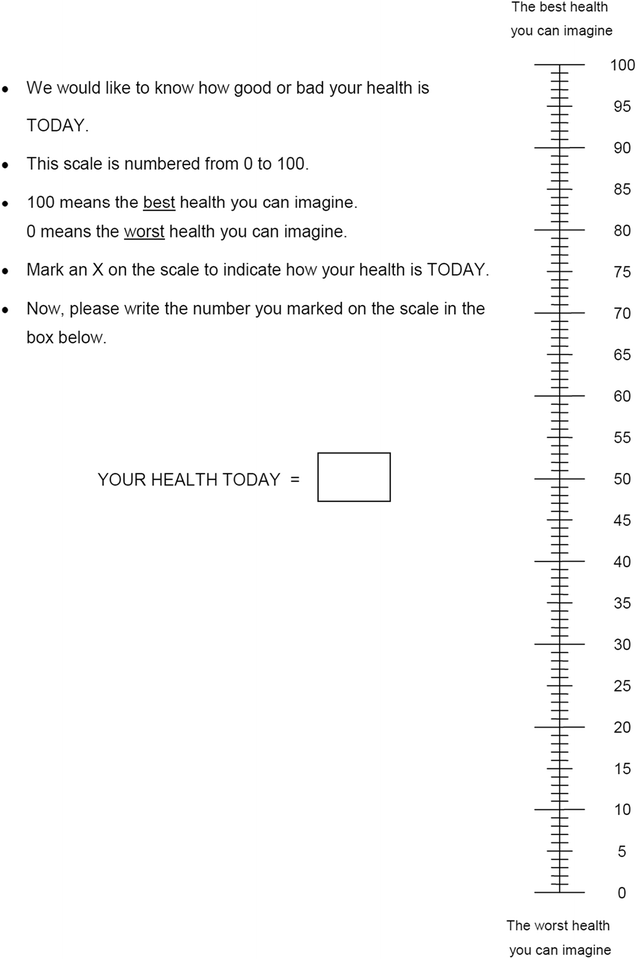

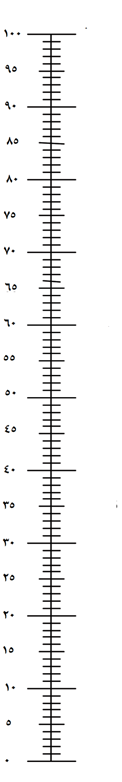


**C) TTO warm up question**

In this part, we asked the respondents to choose between a) and b)

a- 10 years to live in their current state of health.

b- For some time, they exchange these 10 years, in other words, he lives longer, but lives in complete health.

If respondent choose b option, we ask the respondent how much of the current 10 years is willing to scarify? ………

**D) Familiarity with diseases scenario**

**In this section, we asked respondents about their** familiarity with the disease and the history of disease experience by themselves and/or their family member

| **Disease Name** | **Spinal Cord** | **Otitis Media** | **Migraine** | **Eczema** |
| --- | --- | --- | --- | --- |
| **The respondent knows this disease?** Yes: 1 No:2 |  |  |  |  |
| **Has the respondent or another member of her/his household had a history of the disease?(experience)** Yes, Only respondent: 1  Yes, only a member of household: 2 Yes, the respondent and a member of family: 3 No: 4 |  |  |  |  |

Now, the disease scenario presented to respondents. The designed on a card. For example:

| **SPINAL CORD LESION** Spinal cord lesion is subdivided into disease stages as follows:   1. low (acute phase, direct after injury) 2. **low (stable phase)** 3. high (acute phase, direct after injury) 4. high (stable phase)     ***Value now:***  A patient who during the entire year experiences a **stable phase** after a **low spinal cord lesion** (**after** rehabilitation).  Both legs are paralysed.  Other symptoms are: incontinence and, in males, impotence. Also mood changes. | *In what state during the disease?*  ●●●● Unable to walk about  ●●● Many problems with self-care *(washing self, dressing self, eating)*  ●●● Many problems with usual activities *(work, study, housework, family or leisure activities)*  ●● Moderate pain or discomfort  ● A little anxious or depressed  ○ No problems in cognitive functioning *(memory, learning ability, concentration, comprehension)* |
| --- | --- |

Then we asked the respondents to imagine themselves having a target disease and answer the following questions:

| Spinal Cord | Otitis Media | Migraine | Eczema |
| --- | --- | --- | --- |
|  |  |  |  |


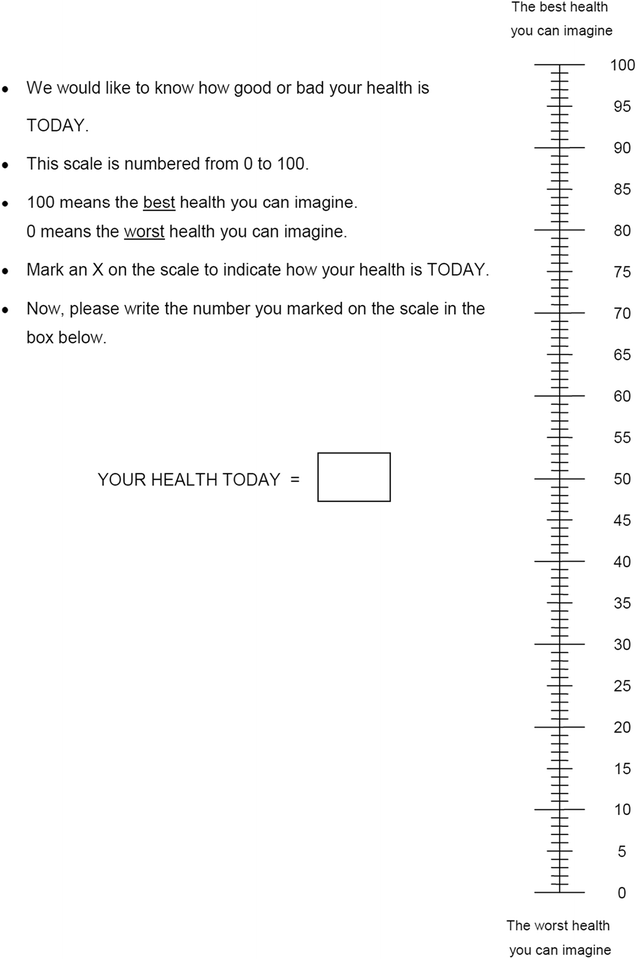

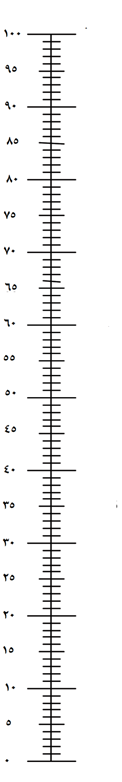

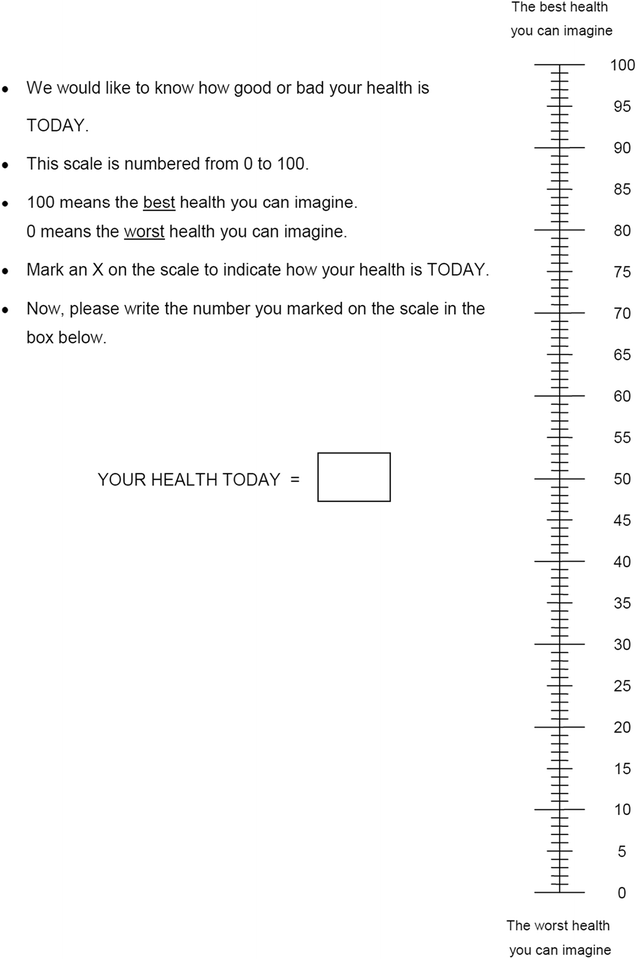

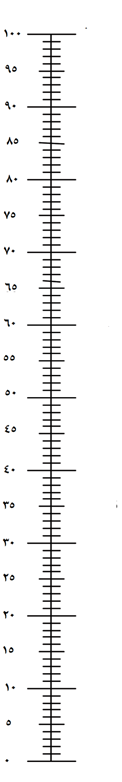

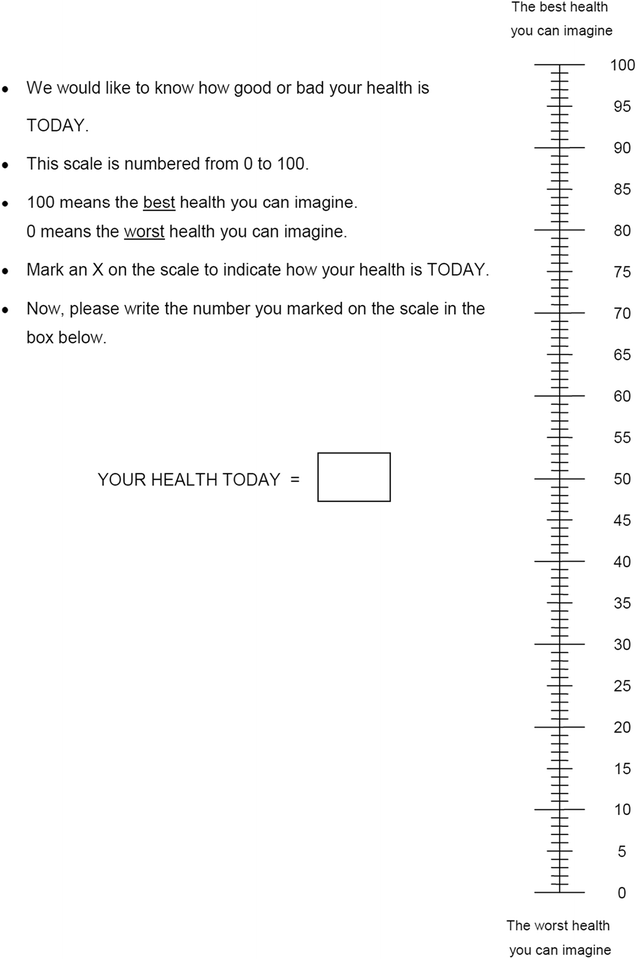

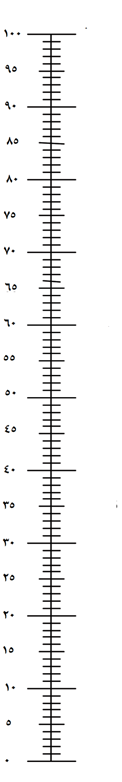

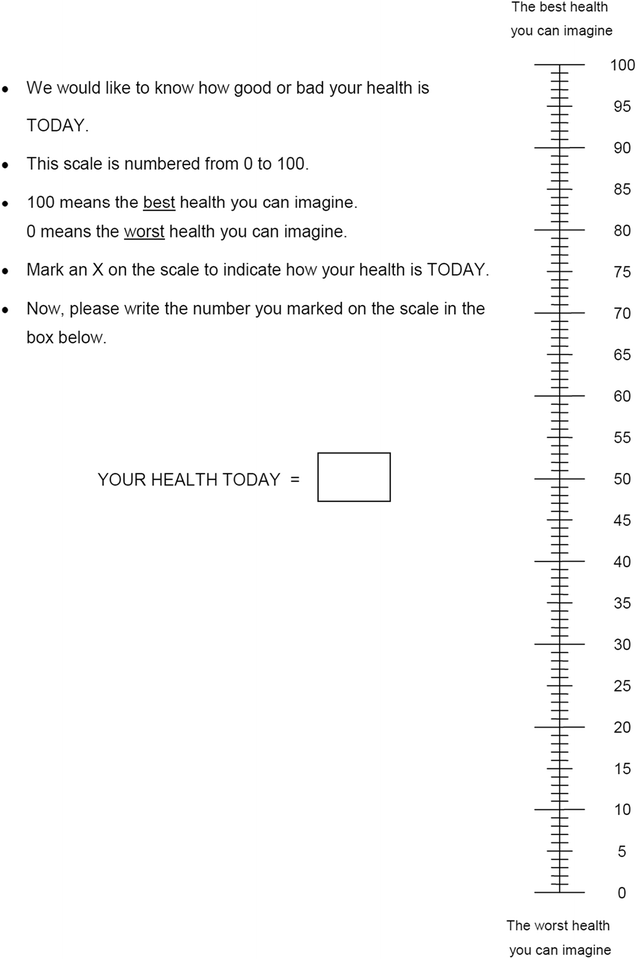

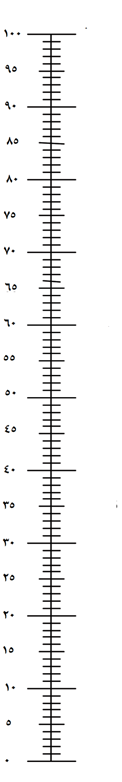


For the TTO measurement, we asked the respondents to imagine themselves having a target disease and state the maximum time that they are willing to sacrifice to avoid being in such a situation for the rest of their life. And asked the based on their age group and the remaing life years according to expected life expectancy.

If a person is not willing to exchange and lose his life, the zero point (0) marked.

If someone exchanging time, it was marked on scale and was written separately.

you must insert the word "zero" in the same bone pan.

| **The remaining life year** | **Age group** |
| --- | --- |
| 0  5  10  15  20  25  30  35  40  45  50  55 | 18-19 |
| 0  5  10  15  20  25  30  35  40  45  50 | 20-29 |
| 0  5  10  15  20  25  30  35  40 | 30-39 |
| 0  5  10  15  20  25  30 | 40-49 |
| 0  5  10  15  20 | 50-59 |
| 0  5  10  15 | 60-69 |
| 0  5  10 | 70-79 |
| 0  5 | 80-84 |
| 0  3 | >85 |

**The reason for not exchanging health with part of life**

In order to complete this section, from respondents with zero answer, we asked the respondent for the most important reason for avoiding to exchange time for that disease,

| **Non- trading resaons /Disease Name** | **Spinal Cord** | **Otitis Media** | **Migraine** | **Eczema** |
| --- | --- | --- | --- | --- |
| no severity of disease |  |  |  |  |
| Religious belief |  |  |  |  |
| because of family and children |  |  |  |  |
| Other reasons |  |  |  |  |

**WTP question**

Finally, the respondents were asked to imagine themselves having a target disease, and state their maximum WTP for a hypotheticals safe treatment without pain and adverse-effect that would cure them of the target disease and he/ she could spend the remaining your life-time in full health, but the treatment was not covered by the government or health insurance and should be paid from his/her own pocket.

Then asked: are you willing to pay even a small amount to get the treatment? Reminding you your budget is limited

If yes, what is the maximum amount which you are willing to pay to completely recover to full health?

1. Please look at this bids and indicate the maximum bid amount that you are willing to pay. Also, consider your Budget constraint. Think about how this additional payment affects your household budget and whether you can take it better

| Less than 333 |
| --- |
| 667 |
| 1,000 |
| 1,333 |
| 1,667 |
| 3,333 |
| 5,000 |
| 6,667 |
| 8,333 |
| 16,667 |
| 25,000 |
| 33,333 |
| 41,667 |
| 50,000 |
| 66,667 |
| 83,333 |
| More than 83,333 |

Bids in US$

1. When the bid amount was stated, the follow up question was asked to elicit the maximum amount.

| **disease scenario** | **the selected bid**  **close –ended question** | **the maximum amount**  **open-ended question** |
| --- | --- | --- |
| **Spinal Cord** |  |  |
| **Otitis Media** |  |  |
| **Migraine** |  |  |
| **Eczema** |  |  |

1. Please indicate the source of your payment.

- Income
- Saving
- Sales of asset
- Borrow
- Others

If the respondents are not willing to pay, please ask the reasons:

- I do not have the financial ability to pay such a fee.
- It is the duty of the government.
- It is a heavy burden for me to pay such expenses.
- Other items
